# Multilevel Selection in the Margins: A Review of Its Representation in Undergraduate Biology Textbooks

**DOI:** 10.1002/ece3.72493

**Published:** 2025-11-16

**Authors:** Charlotte A. Greene, Sarah J. McPeek, Lisa Mitchem, Anne B. Clark, Vincent A. Formica, Conner S. Philson, Robin A. Costello

**Affiliations:** ^1^ Department of Biology University of Virginia Charlottesville Virginia USA; ^2^ Department of Insect Symbiosis Max Planck Institute for Chemical Ecology Jena Germany; ^3^ Department of Biological Sciences Northern Illinois University DeKalb Illinois USA; ^4^ Department of Biological Sciences Binghamton University Binghamton New York USA; ^5^ Department of Biology Swarthmore College Swarthmore Pennsylvania USA; ^6^ Natural Reserve System University of California Santa Barbara California USA; ^7^ Department of Biological Sciences University at Buffalo Buffalo New York USA

**Keywords:** biology education research, evolution education, group selection, levels of selection, multilevel selection, undergraduate biology education

## Abstract

Multilevel selection (MLS) is a foundational framework that expands the concept of natural selection beyond the traditional focus on individuals or genes to larger units, like groups. Despite strong empirical support and applications in a broad range of biological fields, MLS, often referred to as group selection, has a controversial history, which has impeded its wide use and led to incorrect inferences on the sources and strength of selection. Here, we explore how MLS is currently presented to undergraduate biology students by analyzing the content of nine commonly used Evolutionary Biology and Animal Behavior textbooks. We ask whether and how the textbooks describe MLS—as part of the toolbox of evolutionary theory, as a controversial topic, or not at all. Using qualitative textual analysis and descriptive statistics, we found that MLS was mentioned in all of the textbooks analyzed, although usually only in the context of the evolution of social behavior. MLS was generally dismissed as unimportant when compared to individual selection and given less space and fewer examples than kin selection. We highlight the value of a more accurate presentation of MLS and present suggestions for teaching MLS in undergraduate courses based on empirically supported practices in biology education.

## Graphical abstract

This article is a biology education research article that uses a textbook analysis to investigate multilevel selection (MLS) concepts in undergraduate education. We analyze evolution and animal behavior textbooks commonly used in undergraduate classrooms to understand how multilevel selection is discussed in each text, including the number of paragraphs, figures, and examples dedicated to MLS. We find that many undergraduate textbooks do not mention MLS at all, often present MLS as a controversial topic, and provide few examples of MLS.

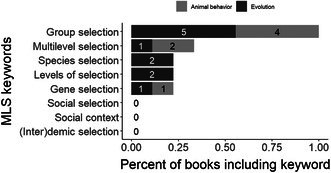

## 1 Introduction

Multilevel selection (MLS) is the theory that natural selection may operate simultaneously at more than one level of biological hierarchy, above and below the level of individuals in a population (e.g., alleles or suites of genes, populations of cells within an individual, social groups within populations). MLS theory traces back to Darwin (1871) and has strong empirical support in evolutionary biology today. Beyond its centrality to evolutionary thinking, MLS has wide‐ranging applications for global challenges in medicine (Lean and Plutynski 2016), pharmacy and drug development (Pepper 2008), epidemiology and public health (Diez Roux 2004; Nonacs and Kapheim 2012; Blackstone et al. 2020), agriculture and landscape ecology (W. M. Muir 1996; Bellamy et al. 2020; Erdos et al. 2024), conservation (Zeller et al. 2017), and developmental psychology (Sober and Wilson 1998). For example, Blackstone et al. (2020) apply multilevel selection theory to SARS‐CoV‐2 infections by considering the viruses inside one host as a structured group within the larger viral population. Using this framework, they explore how both within‐host and between‐host selection could affect viral transmission rates and pathogenicity of SARS‐CoV‐2 under various conditions, allowing researchers to develop more effective vaccines, testing, and contact tracing procedures (Blackstone et al. 2020). However, many evolutionary biologists remain disdainful of MLS (see Eldakar and Wilson 2011), and many more are unaware of its existence and its potential. If we hope to harness MLS to address fundamental and applied problems in evolutionary biology and beyond, we must broaden knowledge of and interest in MLS as an explanatory framework. We believe that this shift begins with accurate and data‐driven teaching of MLS to biology undergraduate and graduate students.

MLS's slow percolation into evolutionary thinking, and consequently, evolutionary biology education, is likely because it remains the subject of one of the most contentious debates in the history of the field. The origin of this debate emerged first around the idea that termite colonies were units of selection just as individuals were (Emerson 1939) and was famously extended by V.C. Wynne‐Edwards' (1962) ideas of groups outcompeting one another. The most famous and also thoroughly debunked example of so‐called “group selection” was that of lemmings hurling themselves off cliffs en masse as a purported form of population control (Chitty 1996). At the peak of the debate in the 1960s, a consensus emerged that the idea of group selection was *theoretically* possible, but highly improbable in the real world (Maynard Smith 1964). Instead, scientists proposed “alternative” theories such as kin selection or individual‐only selection that were more broadly accepted answers for evolutionary processes involving groups (Williams 1966). For many evolutionary biologists, the story of MLS ends here.

However, another school of evolutionary biologists continued to champion MLS thinking. In the 1970s and 1980s, these scientists developed modern theories of MLS that outline how natural selection may operate simultaneously at more than one level of biological hierarchy (Wilson 1975; Heisler and Damuth 1987). This differs from the 1960s, where natural selection was thought to act predominantly on the individual level or predominantly on the group level, but never both simultaneously. These biologists emphasized that selection at these multiple levels may be traced back to fitness consequences for a replicating unit (e.g., gene, individual organism). Modern MLS also includes group selection. Group selection is defined as the differential proliferation of groups of individuals within a population. Because there can be confusion around differentiating group selection and multilevel selection among biologists who do not study modern MLS theory, we would like to clarify that group selection is a term that is encompassed under MLS, in which selection occurs at the level of the group. It is not a synonym for multilevel selection. Modern MLS theory is also not limited to questions of the evolution of selfish and altruistic traits as was the focus of the 60s era debate, but can be applied more generally to a range of biological phenomena including the evolution of complex multicellularity and its breakdown in cancer (e.g., Lean and Plutynski 2016; Capp et al. 2021), patterns of individual plant growth (e.g., Stevens et al. 1995; Donohue 2004), patterns of population growth, (e.g., Wade 1980; McCauley and Wade 1980; Griesemer and Wade 1988, Searcy et al. 2014) the evolution of pathogens, pests, and mutualists in adapting host populations (e.g., Erdos et al. 2024), and the evolution of social and sexual behaviors (e.g., Weinig et al. 2007, Eldakar et al. 2010; Laiolo and Obeso 2012; Royle et al. 2012; Costello et al. 2023; Philson et al. 2025).

While theoretical treatments and empirical support for MLS have accumulated in recent decades, many students receive little exposure to MLS in evolutionary biology education and may not encounter the ideas unless they continue on to graduate training in the field, if they encounter them at all. Additionally, due to this infamous historical split between evolutionary biologists regarding MLS theory, there is often a lack of consensus in the field regarding specific definitions and a clear understanding of multilevel selection, group selection, and more generally, theories of natural selection at multiple levels of biological organization. This lack of consensus makes it all that much more difficult for educators to teach MLS in their classrooms. MLS clearly has tremendous value for understanding evolutionary and environmental change and developing a more complex and nuanced understanding of disease, agriculture, and larger human social processes (Sober and Wilson 1998; Kerr et al. 2006, Hertler et al. 2020). When students are trained in biology without an introduction to MLS, they miss an entire field dedicated to explaining a wide variety of biological phenomena. Because of the plethora of current and future applications of MLS theory to understanding our living world, students are best served with a baseline understanding of MLS theory as part of their training in biology. Furthermore, an informed generation of future researchers can apply and extend MLS to new contexts in biology. Achievement of this goal will be enhanced if MLS is integrated into undergraduate biology so future generations of biologists and evolutionary thinkers can learn to apply MLS as an integral part of modern evolutionary theory, not to dismiss it as a controversial fringe idea.

In this paper, we explore the current status of MLS in undergraduate evolutionary biology education by turning to the platform where young biologists are likely first exposed to the idea of MLS: their textbooks. Textbooks are an essential resource for student learning and provide a curriculum for college and university educators to follow in their course instruction (Skinner and Howes 2013). Authors of biology textbooks, as leading biologists themselves, are not immune to the effects of the historical debate, which may color their presentation of MLS, if they choose to cover the subject at all. Are students encountering the modern theory and the ample supporting empirical evidence for MLS in their biology textbooks? Or are students, even in the face of growing support, still being exposed to MLS as controversial, limited in application, and/or as a failed theory from an earlier era of the field?

We conduct a textbook analysis of five commonly used Evolutionary Biology textbooks for undergraduate students to understand how MLS is being presented to the next generation of biologists and evolutionary thinkers. We also included four widely used texts in Animal Behavior, another place where students may encounter MLS in the context of understanding social behavior. In our textual analysis of commonly used Evolutionary Biology and Animal Behavior textbooks, we address the following three questions:

Question 1: How do Evolution and Animal Behavior textbooks define natural selection? Does their definition accommodate for multilevel selection?

Question 2: Do these texts discuss multilevel selection and how?

Question 3: For textbooks that discuss multilevel selection, how do they mention the controversy surrounding the topic and what level of support do they provide for multilevel selection?

## 2 Materials and Methods

### 2.1 Textbook Selection

We focused our analysis on undergraduate‐level Evolution and Animal Behavior textbooks because these content‐specific introductory‐level courses are where undergraduates are most likely to be first introduced to multilevel selection and are gateways to understanding this concept in future coursework. To identify the most commonly used Evolution and Animal Behavior textbooks in undergraduate courses in the US, we posted on the Society for the Study of Evolution, American Society of Naturalists, Animal Behavior Society, and Society for the Advancement of Biology Education Research listservs to ask members which textbooks they teach in their classrooms. We also contacted Pearson, Norton, Wiley, Oxford University Press, and Robertson Company publishing representatives requesting information on their most frequently purchased Evolution and Animal Behavior textbooks. Lastly, we conducted an Amazon search to identify the textbooks with the most reviews. Our resulting search provided nine textbooks primarily used in introductory‐level Evolution (*n* = 5) and Animal Behavior (*n* = 4) undergraduate courses (Evolution: Bergstrom and Dugatkin 2023; Futuyma and Kirkpatrick 2023; Zimmer and Emlen 2019; Freeman and Herron 2013; Zimmer 2013; Animal Behavior: Nordell and Valone 2020; Dugatkin 2020; Rubenstein 2022; Davies et al. 2012).

### 2.2 Question 1: How Do Evolution and Animal Behavior Textbooks Define Natural Selection? Does Their Definition Accommodate for Multilevel Selection?

To analyze how Evolution and Animal Behavior textbooks define natural selection, we utilized a deductive thematic analysis approach to group definitions into predefined categories (Saldaña 2013). We used a deductive approach as our analysis had a specific goal to identify if and how definitions of natural selection accommodate multilevel and group selection. We determined that definitions would accommodate multilevel and group selection within natural selection if the definition did not specifically reference “individual” within the definition.

We first pulled natural selection definitions from the textbook glossary, when present, or the text itself, when not present. Two researchers (C.A.G. and S.J.M.) independently read through all definitions and assigned definitions to one of two categories: Definitions that explicitly included “individuals” as the unit of selection, and definitions that did not specify the unit of selection. After independently categorizing definitions, the two researchers met to ensure alignment and agreement.

### 2.3 Question 2: Do These Texts Discuss Multilevel Selection and How?

We utilized a mixed‐methods approach (Warfa 2016) to analyze whether and how multilevel selection is presented in introductory Evolution and Animal Behavior textbooks. We first analyzed whether multilevel selection was defined using a set of keywords related to multilevel selection. These keywords included: multilevel (or multilevel) selection, group selection, social selection, (inter)demic selection, species selection, social (or group) context/group context, gene selection, and levels of selection. For textbooks that included one or more of these keywords, we qualitatively analyzed how they defined multilevel selection (see below) to ask (Question 2A) *How do textbooks define multilevel selection?* We further analyzed the overarching chapter in which multilevel selection was presented, the amount of space dedicated to the topic of multilevel selection, and the diversity of examples provided about multilevel selection to answer the following subquestions: (Question 2B) *In what context is multilevel selection mentioned?*, (Question 2C) *How much space is dedicated to multilevel selection topics?*, (Question 2D) *What examples are used to illustrate multilevel selection topics?*, and (Question 2E) *For textbooks that discuss multilevel selection, how is kin selection discussed in comparison to multilevel selection?*

To qualitatively assess how textbooks define multilevel selection (Question 2A), we pulled definitions for multilevel selection and group selection from the textbook glossary, when present, or from the text itself. We focused our analysis on multilevel and group selection because these terms were the most commonly used across textbooks based on our quantitative analysis (see Results). For this analysis, researchers joined multilevel selection and group selection definitions since very few textbooks contained the term multilevel selection. Additionally, these two terms were used interchangeably. Therefore, researchers coded definitions at the textbook level, with eight total definitions from the eight textbooks used in this analysis. Two researchers (R.A.C. and L.M.) then coded the definitions using an inductive thematic analysis approach, rather than using a predetermined list of codes (Saldaña 2013). The two researchers first independently read through all the definitions of multilevel and group selection and identified key emerging themes (i.e., codes). The researchers then met to develop a consensus list of codes to use as their codebook. The final codebook included five codes, and the two researchers used axial coding to group and abstract the codes into two major categories (Saldaña 2013). These two categories included definitions of multilevel selection that mentioned (1) simultaneous individual‐ and group‐level selection and (2) differential survival and productivity of groups (Figure 2). The researchers then independently categorized each definition according to the codebook using all appropriate codes. Intercoder reliability was calculated to measure the degree of alignment between the two researchers' use of the codebook. To establish trustworthiness for our analysis, the researchers coded to consensus on all definitions. Initial agreement was 76%, and all definitions with mismatched code designations between researchers were discussed until the two researchers reached 100% agreement.

We collected data on the presence/absence of each keyword related to multilevel selection in the textbook index, the chapter in which the keyword was discussed (Question 2B), the number of paragraphs dedicated to the keyword (Question 2C), and any examples used to explain the keyword (Question 2D). Each of the nine textbooks was sampled by two independent researchers. When data between the textbook samples did not align, two researchers (C.A.G. and S.J.M.) reexamined the text to identify discrepancies and decide how to resolve differences in data collection methods. For example, we decided through this process not to include information in Boxes as part of the paragraph count but to include examples in Boxes as part of our examples dataset. We report summary statistics for the above‐mentioned variables. For Questions 2B–2E, we combined any textbook content using either the keywords “multilevel selection” and “group selection” under the single umbrella topic “multilevel selection” as qualitative coding of definitions of multilevel selection revealed little distinction among how textbooks described multilevel selection and group selection (i.e., these terms are interchangeable across books).

We collected examples related to three of our keywords: “multilevel selection,” “group selection,” and “kin selection,” focusing only on examples in which that term was specifically referenced. For each example, we noted the keyword the example was associated with, the taxon, the context of the example (i.e., cooperative breeding, population regulation), whether the example was fictitious or real, and any literature cited by the text relating to that example. One researcher (SM) then reviewed all MLS examples and grouped them into categories based on the broad taxonomic group (i.e., birds, fish, insects, mammals), the context described by the example (i.e., population regulation, cooperative behavior, evolution of multicellularity), and whether the example supported, opposed, or took no position on the veracity of MLS. An additional researcher (CG) reviewed these categorizations and discussed any disagreements over appropriate categorization until a consensus was reached.

We also assessed how kin selection is discussed in comparison with multilevel selection. Kin selection is often framed as an alternative explanation to MLS, despite the two being complementary approaches quantifying different concepts: MLS quantifies the direction of evolutionary change and kin selection quantifies optimality models. We assessed how kin selection is discussed in comparison with MLS by comparing the frequency, space used, and diversity of examples between the multilevel selection keywords (both multilevel selection and group selection) and keywords related to kin selection (i.e., kin selection and inclusive fitness) (Question 2E). Due to our sample size of nine textbooks, we could not make statistical comparisons between how textbooks discuss multilevel selection and kin selection. Instead, we provide descriptive statistics for the two concepts.

### 2.4 Question 3: For Textbooks That Discuss Multilevel Selection, How Do They Mention the Controversy Surrounding Multilevel Selection and What Level of Support Do They Provide for Multilevel Selection?

A similar inductive thematic coding process was used to analyze discussions of controversy about multilevel selection. The researchers identified controversial passages as any statement that used argumentative words including, “argued/argument,” “controversial,” “critical,” “debate,” and “reject/accept.” Two researchers (R.A.C. and L.M.) independently reviewed the first 10 controversial passages to identify codes and then met to create the codebook. The final codebook included eight different codes. The researchers used axial coding to group these codes into three different overarching categories: Passages that provided (1) full support, (2) theoretical support, and (3) no support for evolution by multilevel selection (Figure 6). The researchers then used the codebook to independently code all 32 controversy passages, calculated intercoder reliability (initial agreement was 76%), and came to 100% agreement on any controversial passages with mismatched codes. Note that each controversial passage could be assigned to multiple codes.

We measured the frequency of passages in each category and found that controversial passages most commonly did not support multilevel selection and argued that individual selection provides a better explanation (see Results below). Using the same process described above, the same two researchers created another codebook to then categorize these passages into different reasons provided for why multilevel selection is better explained by individual selection. This codebook included six different codes (Figure 7). Their initial agreement was 63%, and the two researchers reached 100% agreement for all passages with mismatched codes.

### 2.5 Positionality Statement

Science is a human enterprise and subject to the biases of the authorship team (Secules et al. 2021). In an effort to increase transparency, we acknowledge our past experiences that inform our research approaches and conclusions. Our scholarly expertise includes evolutionary biology, behavioral ecology, animal behavior, and biology education research. Many of our past and current research projects focus on multilevel selection, and we understand multilevel selection as selection that occurs simultaneously at multiple levels. We are experts in both quantitative and qualitative methodological approaches, and we leverage our expertise to understand how introductory Evolution and Animal Behavior textbooks present multilevel selection.

## 3 Results

### 3.1 Question 1: How Do Evolution and Animal Behavior Textbooks Define Natural Selection? Does Their Definition Accommodate Multilevel Selection?

Before we examined treatments of multilevel selection in these textbooks, we first explored how textbooks introduce the concept of natural selection, as this definition determines whether multilevel processes can be included within a broad natural selection framework. Out of nine introductory Evolution and Animal Behavior textbooks, only three did not use the term “individual” when describing the unit of selection, meaning that six of the nine textbooks specified that natural selection occurs at the level of individuals in a population.

### 3.2 Question 2: Do These Texts Discuss Multilevel Selection and How?

**FIGURE 1:**
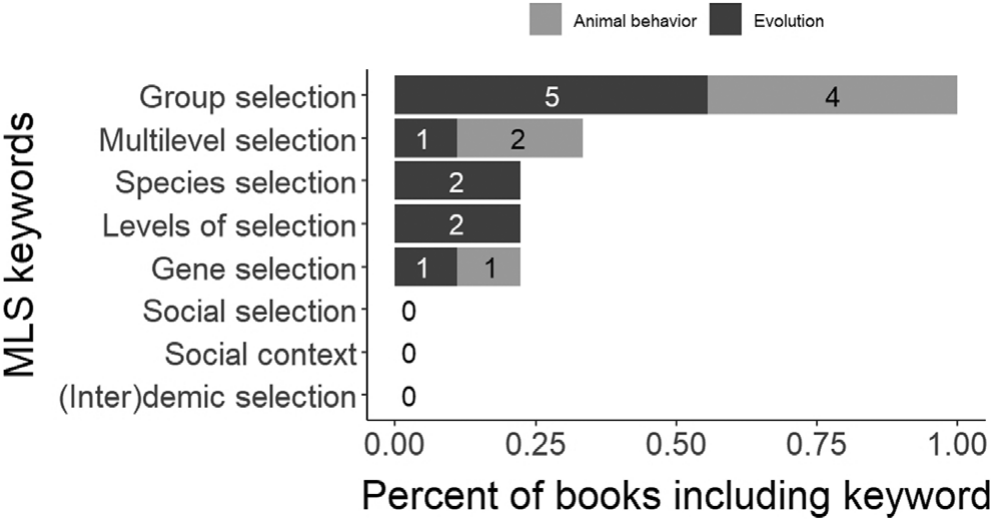
Number of books that included each keyword in their textbook index. *N* = 9 textbooks, 5 Evolution texts, and 4 Animal Behavior texts.

Every textbook used the term “group selection”, but only three out of the nine textbooks used the term “multilevel selection” (Figure 1). Broken up by textbook type, two of the four Animal Behavior textbooks and one of the five Evolution textbooks explicitly used the term “multilevel selection”. Our other keywords (i.e., social selection, (inter)demic selection, species selection, social (or group) context, gene selection, and levels of selection) were mentioned in two or fewer textbooks. As such, we focus the remainder of our analyses on content associated with the terms “multilevel selection” and “group selection.”

#### 3.2.1 Question 2A: How Do Textbooks Define Multilevel Selection?

**FIGURE 2:**
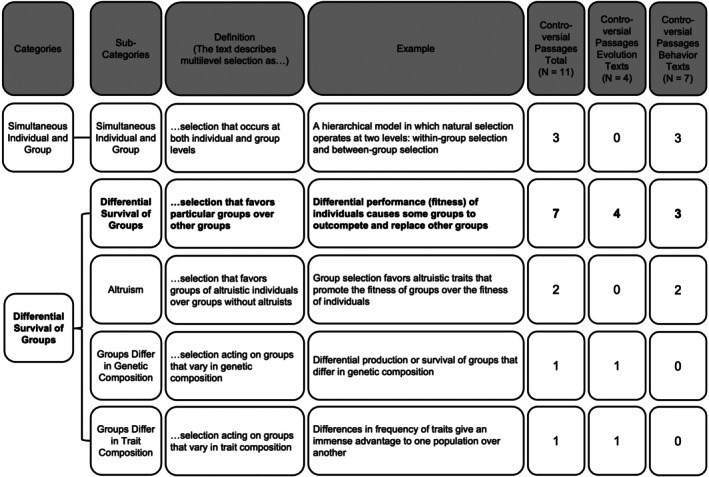
Qualitative codebook (i.e., a list of categories and subcategories developed to describe emergent themes, Saldaña 2013) used to organize themes presented in multilevel selection definitions in textbooks. The most frequently used definition is bolded.

We qualitatively coded definitions provided for the two most commonly mentioned keywords related to multilevel selection: “group selection” and “multilevel selection”. We did not find a distinction between definitions of multilevel and group selection across texts. Both multilevel and group selection were defined as selection acting on groups and as selection acting at both the individual and group levels. We therefore analyzed definitions of multilevel and group selection together, which we hereafter refer to as simply multilevel selection. We found that the majority of textbooks (seven out of nine) defined multilevel selection in terms of the differential survival of groups (Figure 2). Three textbooks, all Animal Behavior texts, described multilevel selection as selection that occurs at both the individual and group levels (Figure 2). Figure S1 describes how each textbook in our sample defined multilevel selection.

For the remainder of these results, we will combine multilevel selection and group selection content under the broader umbrella of multilevel selection, as our definition coding revealed that books referred to substantively similar concepts using either term.

#### 3.2.2 Question 2B: In What Context Is Multilevel Selection Mentioned?

Two of the nine textbooks (22% of the sample, one Evolution text and one Animal Behavior text) discussed multilevel selection in the context of discussions of natural selection. The remaining seven textbooks (78% of the sample) discussed multilevel selection in chapters explicitly focused on topics of social behavior, altruism, and the evolution of cooperation.

#### 3.2.3 Question 2C: How Much Space Is Dedicated to Multilevel Selection Topics?

**FIGURE 3:**
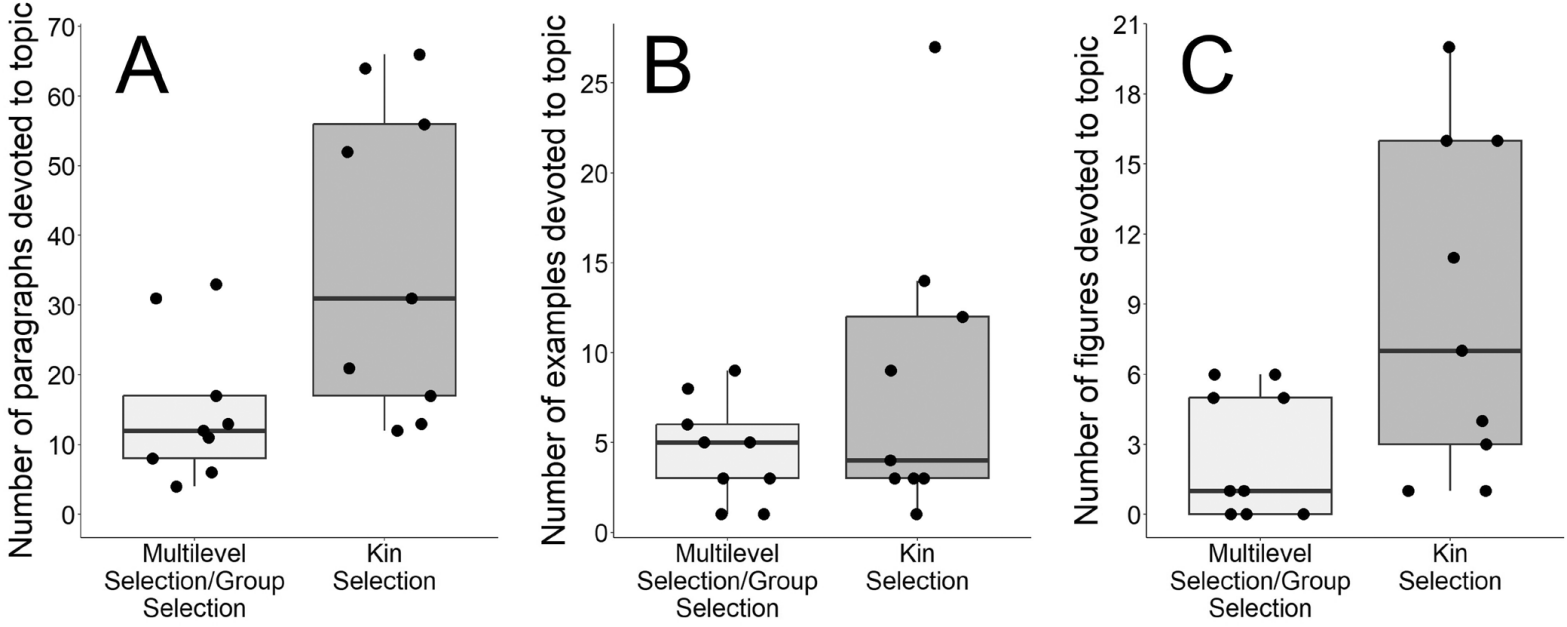
Quantitative assessments of multilevel selection and kin selection coverage across the nine textbooks in terms of number of paragraphs (A) number of examples (B) and number of figures (C) associated with each topic. Points are individual books.

Texts varied widely in the number of paragraphs, examples, and figures dedicated to multilevel selection concepts (Figure 3). Books spent an average of 15 paragraphs covering MLS topics, with two devoting between 20 and 35 paragraphs to the subject, and three only dedicating four to eight paragraphs to the subject (Figure 3A). A similar trend of varying coverage emerged in the number of examples that books used to illustrate multilevel selection and the number of accompanying figures (Figure 3B,C). Books provided a mean of six different examples of multilevel selection concepts and a mean of five figures to illustrate those examples. Three of the nine books provided zero to one figures to illustrate multilevel selection concepts.

#### 3.2.4 Question 2D: What Examples Are Used to Illustrate Multilevel Selection Topics?

**FIGURE 4:**
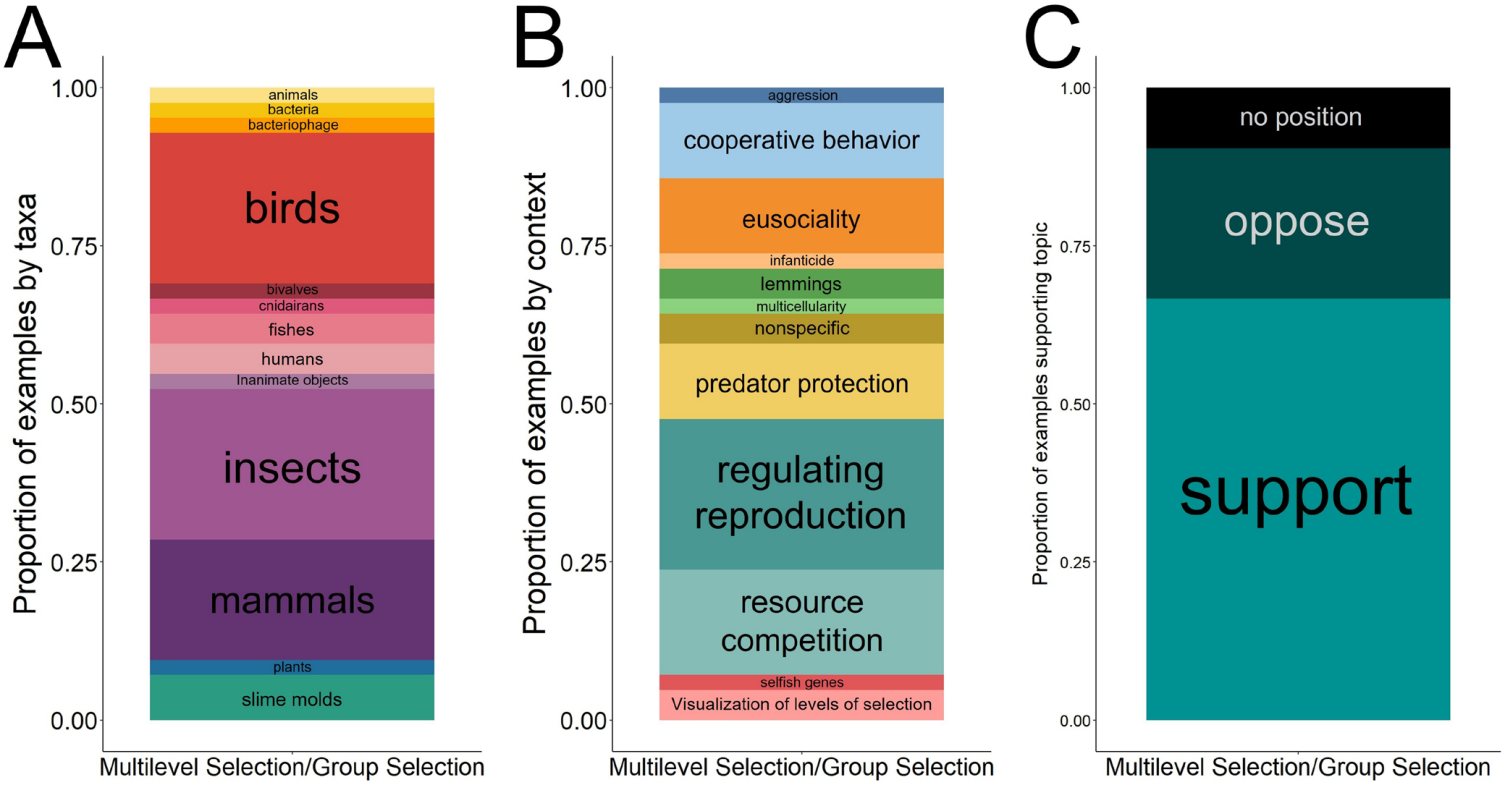
Taxonomic coverage (A), contextual breadth (B), and level of support for MLS (C) encompassed in the examples that textbooks used to discuss MLS concepts.

The taxonomic diversity of the examples used by textbooks to illustrate multilevel selection (Figure 4) varied among the textbooks we examined. Two‐thirds of examples used to illustrate multilevel selection occurred in only three taxa: birds (10 examples), insects (nine examples), and nonhuman mammals (eight examples, Figure 4A). The majority of examples within insects (eight of nine) were of eusocial insects. Slime molds were the fourth most commonly cited taxon with a total of three examples across texts. All other taxa received fewer than three examples.

The range of natural contexts to which multilevel selection concepts were applied was similarly limited (Figure 4B). The two most commonly referenced contexts were regulating reproduction (ten examples, i.e., cooperative breeding, self‐limiting clutch size) and resource competition (seven examples, i.e., competition for mates, food, other resources). Three other contexts received greater than four instances of coverage: cooperative behavior (i.e., hunting), predator protection (i.e., schooling behavior), and eusociality. 60% of all examples focused on behavioral traits in social animals. Two texts referred to the faulty logic of group selection as a means of population regulation using the infamous case of Alaskan lemmings (Chitty 1996).

Approximately two‐thirds (66%) of the examples provided for multilevel selection were used to support the existence of multilevel selection, 24% of examples were used to disprove multilevel selection, and 10% of examples did not state an explicit position on the biological reality of multilevel selection concepts (Figure 4C).

**FIGURE 5:**
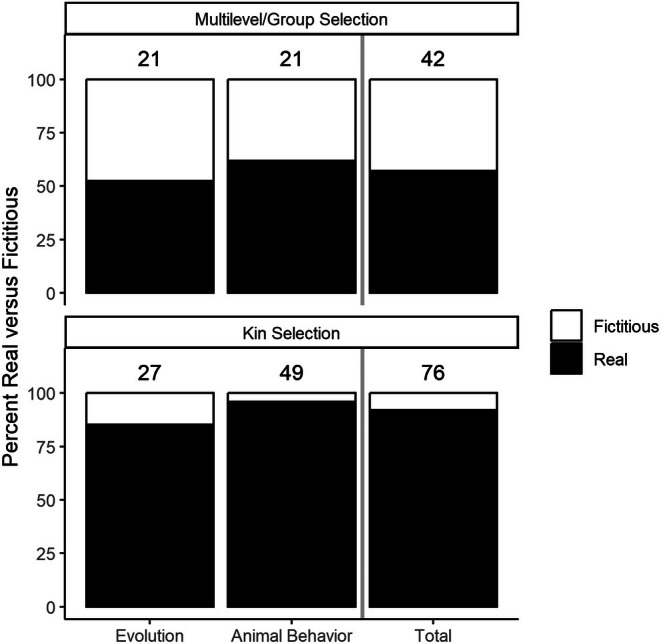
Proportion of fictitious versus real‐world examples given when explaining or justifying MLS and kin selection. Texts used a much larger proportion of real‐world examples for kin selection than they did for multilevel selection.

Across all examples from all nine books, 57% of these examples were supported by real‐world data, and 43% of these examples were fictitious in nature (Figure 5). This proportion of real to fictional examples was similar across Evolution and Animal Behavior texts.

#### 3.2.5 Question 2E: For Textbooks That Discuss Multilevel Selection, How Is Kin Selection Discussed in Comparison to Multilevel Selection?

All nine textbooks discussed the concept of kin selection. Eight of the nine textbooks mentioned “inclusive fitness” and seven of the nine textbooks mentioned “kin selection.” While two out of the nine textbooks did not mention the term “kin selection,” these two texts did mention the term “kin” and discussed the importance of kin in natural selection. Kin selection was introduced in chapters on the evolution of social behavior, frequently presented alongside multilevel selection. Specifically, six of the nine textbooks introduced MLS and kin selection in the same chapter. Across textbooks, there were more paragraphs, more examples, and more figures dedicated to explaining kin selection and related concepts than to multilevel selection (Figure 3). Furthermore, over 80% of examples for kin selection were based on real‐world examples, while only around 50% of examples for MLS were based on real‐world examples (Figure 5). Figure S2 lists the ratio of fictitious to real‐world examples for every textbook included in our sample.

### 3.3 Question 3: For Textbooks That Discuss Multilevel Selection, How Do They Mention the Controversy Surrounding the Topic and What Level of Support Do They Provide for Multilevel Selection?

**FIGURE 6:**
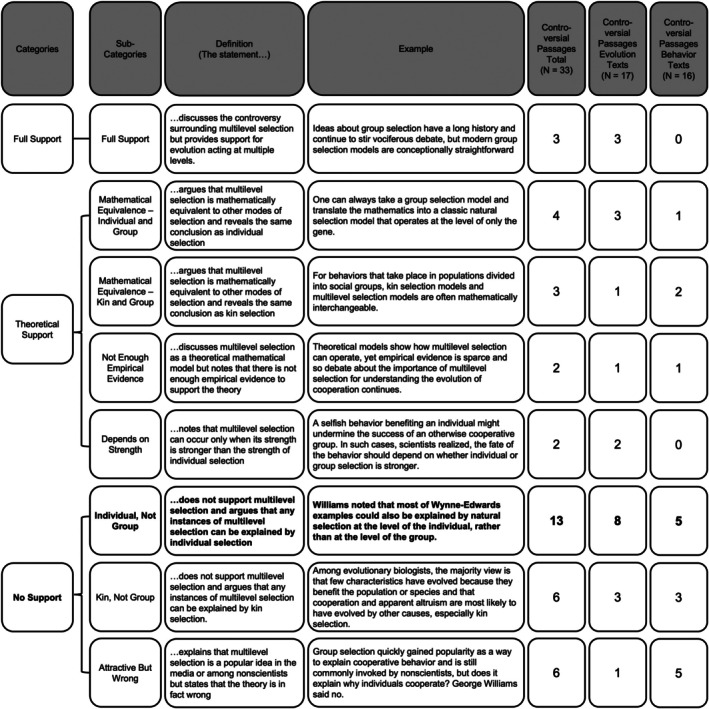
Codebook of how passages discuss the controversy surrounding multilevel selection. The most common discussion around the controversy is bolded.

We found that all texts included passages discussing the controversy surrounding multilevel selection. In total, we found 33 different passages that mentioned this controversy and thematically coded these passages for the conclusions they draw (Figure 6). We found that 9% of the passages provided full support for selection acting at multiple levels of biological organization. 30% of passages discussed how multilevel selection had theoretical support but cautioned that either there was not much empirical support or that this theoretical support was mathematically equivalent to individual selection or kin selection. The majority of passages (67%) did not support multilevel selection. These passages argued that individual and/or kin selection provided better explanations and that multilevel selection was an attractive but inaccurate theory. Nine of the controversial passages (27%, 4 from Evolution texts and 5 from Animal Behavior texts) discussed multilevel selection in the context of the evolution of altruism. Figure S3 describes how each textbook in our sample discusses the controversy surrounding multilevel selection.

**FIGURE 7:**
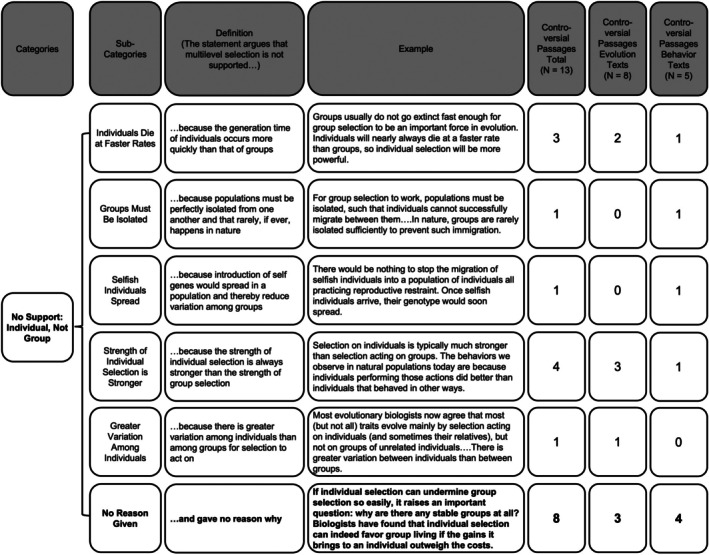
Codebook of the different reasons given for why selection occurs at the individual level but not at the group level. The most common reason is bolded.

Thirty‐nine percent of passages that discussed multilevel selection as controversial conclude that multilevel selection is not supported models because all instances of multilevel selection can be explained instead by selection occurring only at the level of the individual. Given this focus across texts on individual selection over multilevel selection, we coded the reasons provided for why individual selection provides a better model of selection than does multilevel selection (Figure 7). We found that the majority of passages (62%) arguing that individual selection provides a better explanation for multilevel selection did not provide an explanation. Thirty‐one percent of passages argued that the strength of individual selection is always stronger than group selection, and 23% described how individuals die at faster rates than groups, making individual selection a stronger force of evolutionary change than group selection. Figure S4 describes the reason each textbook gives for why selection occurs at the individual, not group, level.

## 4 Discussion

Our analysis of commonly used Evolution and Animal Behavior textbooks revealed that most texts devote little space to MLS and further dismiss MLS as an unimportant or incorrect evolutionary framework. Two‐thirds of all textbooks in this analysis defined natural selection as a process that occurred at the level of the individual, precluding the possibility of selection at multiple levels of biological organization. Although all texts mentioned MLS, they typically dedicated less space to and provided fewer examples about MLS when compared to kin selection, an evolutionary framework that also explores the evolution of group‐level processes among genetically related individuals. Furthermore, the provided examples of MLS were often fictional and discussed cases that did not support selection occurring at multiple levels. Finally, all texts referenced the debate surrounding group selection from the 1960s and commonly dismissed MLS as an unnecessary framework that can easily be explained by individual or kin selection.

Taken together, our findings suggest that undergraduate biology students are taught to reject a multilevel understanding of evolutionary processes in the majority of Evolution and Animal Behavior textbooks. However, this dismissal of MLS is at odds with broad empirical support that advances our understanding of the evolution of key processes, including multicellularity (e.g., Lean and Plutynski 2016; Capp et al. 2021), pathogenicity (Diez Roux 2004; Nonacs and Kapheim 2012; Blackstone et al. 2020), and social behaviors (e.g., Eldakar et al. 2010; Laiolo and Obeso 2012; Royle et al. 2012; Costello et al. 2023; Philson et al. 2025). We suggest that authors and publishers of textbooks update their treatment of MLS to encompass the field's current understanding of this valuable body of evidence. Here, we provide suggestions (listed in bold) for how instructors can overcome the current shortcomings of commonly used textbooks and employ evidence‐based pedagogical practices to teach MLS in their undergraduate courses.

### 4.1 Define Natural Selection in a Way That Accommodates Multilevel Selection

All texts except for one limited their definition of natural selection to the level of the individual. This narrow definition does not permit the possibility of selection acting on multiple levels and sets the stage for the immediate rejection of MLS when the topic is eventually introduced in the course. To counter this narrative, instructors can broaden the definition of natural selection to include selection at the levels of genes, genomes, cells, individuals, mutualistic partners, groups, or taxa. This definition need not be a major departure from more commonly used definitions. For example, “any consistent difference in fitness among phenotypically different classes of biological entities” is a broadened definition similar to the one provided by Futuyma and Kirkpatrick (2023) that better reflects a modern understanding of natural selection (Okasha 2006) while remaining specific and faithful to the original ideas. Furthermore, by clarifying that selection can act at multiple levels when first defining natural selection, instructors of Evolution and Animal Behavior can introduce the concept of MLS much earlier in their course, instead of relegating MLS to a special topic that could create confusion later in the course.

### 4.2 Clarify Terminology Associated With MLS

Although all textbooks used the term “group selection” to refer to MLS, the texts also used “multilevel selection,” “levels of selection,” “species selection,” and “gene selection.” This widely variable terminology reflects the MLS scholarship (Okasha 2006), which also includes other MLS‐related terms not found in the textbooks, such as “social selection” (Wolf et al. 1999; McGlothlin et al. 2010; Formica et al. 2011), “social context” (Heisler and Damuth 1987; Goodnight et al. 1992), and “(inter)demic selection” (Wilson 1979). Not only do texts use many different terms to describe MLS, but they also define MLS in different ways. Although most texts defined MLS as the differential survival and proliferation of groups, several texts defined MLS as selection acting simultaneously at multiple levels of biological organization. Some texts used different terms for these two different definitions (i.e., they referred to the differential survival of groups as “group selection” and selection operating at multiple levels as “multilevel selection”), but this distinction was not consistently applied across texts. The confusion created by this inconsistent use of many different terms was noted in the textbooks included in this study, as Freeman and Herron (2013) explain, “Biologists vary in the words they use to describe the mechanisms of evolution that operate in populations structured into groups (West et al. 2007). This can give the impression of more disagreement over the process than actually exists (Lion et al. 2011; Marshall 2011).” There is a clear need for MLS researchers to reach consensus on which terms to use to describe selection acting on multiple levels and how to define those terms (Okasha 2006, 2010a). For more in‐depth discussion, Okasha's book, *Evolution and the Levels of Selection* Okasha (2006), offers a rich and nuanced review of many of the terms and theories associated with MLS. We strongly support the specific clarification and definitions of terms associated with MLS in this book and recommend it as a starting point for educators looking to implement MLS into their curriculum. With clarified terminology, educational resources and instructors can more easily communicate MLS to undergraduate biology students.

### 4.3 Present Applications of MLS Outside of the Evolution of Social Behavior

The majority of texts only discussed MLS when explaining the evolution of social behavior in animal species. They commonly introduced MLS when presenting the paradox of the evolution of cooperative behaviors (e.g., cooperative breeding, self‐limiting reproductive output, self‐limiting resource consumption, group hunting, schooling) and social systems (e.g., eusociality). In these contexts, multilevel selection was evoked to explain how selection on cooperative behaviors at the level of the group can counter individual‐level costs to altruism. However, multilevel selection was most often dismissed during discussions of the evolution of social behaviors in favor of kin selection despite the two being complementary, not opposing, ideas (Queller 1992; Bijma and Wade 2008; Bijma and Aanen 2010). This kin selection favoritism is illustrated by the limited amount of space devoted to MLS compared to kin selection. For instance, kin selection had 76 examples across the 9 books (with over 90% being real‐world examples) while multilevel selection only had 42 examples (with only half being real‐world examples) (Figure 5).

Mirroring the limited context in which texts discussed MLS, the examples provided about MLS were primarily restricted to social animals, including birds, eusocial insects, and nonhuman mammals. This limited focus on social animals does not reflect the broad empirical support for MLS that exists across taxa (see Marín et al. 2025 in this Special Issue for a comprehensive description of empirical support for MLS and the breakdown of this support by taxa). However, not all textbooks restricted their examples to social animals. For example, Freeman and Herron (2013) provide in‐depth discussions of the experimental evolution of cooperation in bacteria (Chuang et al. 2009) and leaf area in plants (Stevens et al. 1995) resulting from group‐level selection. By highlighting examples beyond altruism and complex social behaviors, textbooks and instructors can demonstrate that MLS is more than a solution for apparent paradoxes and complex situations, but rather is a tool for understanding a vast array of evolutionary phenomena.

Highlighting a variety of contexts in which MLS is relevant beyond understanding the evolution of social behavior is also especially important for engaging biology students with widely varying interests and career goals. The vast majority of undergraduate biology students are interested in pursuing careers in the health sciences (McCartney et al. 2025); however, biology students are also interested in careers in agriculture, education, conservation, and research. As such, instructors can incorporate examples of how researchers employ multilevel selection to understand the evolution of cancers (e.g., Bentley et al. 2022; Capp et al. 2021; Lean and Plutynski 2016) and pathogens, including SARS‐CoV‐2 (Blackstone et al. 2020), and examples of how applying multilevel selection to livestock and crops can increase agricultural yields (e.g., Ellen et al. 2008; W. M. Muir 1996, 2005; Wade, Bijma, et al. 2010) to engage students with career aspirations in the health sciences and agriculture, respectively. Previous work has noted that students struggle to find the personal relevance of evolutionary biology (Hanisch and Eirdosh 2020) and found that students engage more in learning course content when the content is relevant to their personal and professional interests (Schroeder et al. 2007). Presenting MLS outside of the context of the evolution of social behavior will subsequently allow students that study other subfields of biology to engage more strongly with MLS. For example, a student that is interested in pursuing medical school may be more invested in MLS theory if educators teach an example of how MLS is used to study infectious disease transmission or cancer progression. Therefore, we believe presenting a variety of different examples of MLS that span various subfields is an important step in teaching the next generation of biologists to apply MLS in their respective fields.

### 4.4 Discuss How MLS Applies to Real‐World Contexts

Nearly half of the examples about MLS provided in textbooks were fictitious (i.e., not published empirical examples of MLS). This reliance on hypothetical examples contrasts starkly with the large proportion of empirical examples used to explain kin selection. Using fictitious examples to explain MLS likely stems from the origins of the theory of MLS, which relied heavily on theoretical examples. For example, V. C. Wynne‐Edwards (1962) described fictitious groups of birds consuming food at different rates to argue for selection acting at the group level, and David Sloan Wilson (1975) developed his model of multilevel selection using an abstracted population of cooperative and selfish individuals. While describing hypothetical examples is a useful heuristic tool to explain unintuitive and difficult concepts to students, discussing real‐world examples greatly improves student learning and promotes student interest in science (Schroeder et al. 2007; Kjelvik and Schultheis 2019; Schultheis et al. 2023). For example, work on Data Nuggets, a compendium of quantitative biology activities that features data collected by scientists, found that utilizing authentic examples and data in undergraduate biology courses increases student engagement in course content, their self‐efficacy in their scientific skills, and their interest in science careers (Kjelvik and Schultheis 2019; Schultheis et al. 2023). Additional research found that curricula focused on the real‐world consequences of biodiversity improved student learning of course content compared to curricula focused on the concept of biodiversity itself (Timmerman et al. 2008; Ballen and Greene 2017). The MLS Initiative is currently developing a repository of examples and course activities to aid instructors in incorporating more real‐world examples of MLS that have relevance to students' lives in their courses (ProSocial World, n.d.). Discussing MLS in courses with empirical data from a wide variety of systems can move students' understanding of the field beyond a theoretical debate that can be easily dismissed as irrelevant and towards a modern evolutionary theory necessary for making sense of real‐world phenomena.

### 4.5 Teach MLS as an Important Framework in Evolution, Despite History of Controversy

Despite the broad empirical support for MLS, all the textbooks in our analysis discussed the controversy surrounding MLS. In addition to the historical debate of the 1960s over whether selection can act at the level of groups (Wynne‐Edwards 1962; Williams 1966), multilevel selection remains a subject of contentious debate in evolutionary biology fields (Okasha 2006; Maher 2009; Wild et al. 2009; Okasha 2010b; Wade, Wilson, et al. 2010; Wild et al. 2010; Eldakar and Wilson 2011; Wilson 2008; Gardner 2015; Goodnight 2015). We encourage instructors to provide the accurate context of multilevel selection alongside the ample empirical evidence across disciplines (cancer: Lean and Plutynski 2016; Capp et al. 2021; plant growth: Stevens et al. 1995; Donohue 2004; population growth: Wade and McCauley 1980; Searcy et al. 2014; evolution of pathogens, pests, and mutualists: Erdos et al. 2024; social and sexual behaviors: Eldakar et al. 2010; Laiolo and Obeso 2012; Royle et al. 2012; Costello et al. 2023; Philson et al. 2025). Best practices for teaching actively debated scientific topics within the scientific community are currently lacking, although studies have investigated approaches to teaching scientific issues that are controversial in the public sphere, including climate change and vaccinations (Oulton et al. 2004; Lobato and Zimmerman 2019; Beniermann et al. 2021). Open questions revolve around the impact of presenting the continued debate surrounding MLS—will the scientific debate motivate students to engage in the topic and develop critical thinking skills or demotivate students by exposing them to philosophical and semantic arguments? Regardless of how instructors discuss the MLS controversy, we urge instructors to emphasize that multilevel selection is a fundamental evolutionary theory with broad empirical support. By teaching multilevel selection with its proper evidence, multilevel selection will no longer be viewed as controversial to students, but as another tool in their evolutionary toolkit to understand the natural world.

### 4.6 Apply Evidence‐Based Teaching Practices

Textbooks cannot be the only source of MLS education, especially given the general lack of coverage of these topics. Drawing from the field of biology education research, we recommend employing evidence‐based pedagogical practices when teaching MLS in undergraduate biology courses (Tanner 2013). Specifically, we recommend incorporating active learning strategies that have proven to increase student performance and promote equitable learning (Freeman et al. 2014; Theobald et al. 2020). In particular, we recommend that instructors incorporate asking open‐ended questions related to MLS in their lectures, and provide a variety of active learning strategies for students to answer those questions, such as think‐pair‐share, written reflection, or concept mapping (Tanner 2013). Proposing open‐ended questions without a specific correct answer helps build student confidence and thereby fosters an inclusive environment in which all students may feel comfortable enough to ask questions without judgment (Allen and Tanner 2002; Crowe et al. 2008). Furthermore, as mentioned above, integrating real‐world examples into undergraduate biology courses can increase student interest in science and improve student learning (Schroeder et al. 2007; Kjelvik and Schultheis 2019; Schultheis et al. 2023). We find an example of multilevel selection in chickens leading to increased production and decreased mortality and cannibalism (W. M. Muir 1996; Craig and Muir 1996) a compelling and easy‐to‐explain example. Presenting the data from these papers and encouraging students to draw their own inferences can be a powerful and memorable classroom activity, while also touching on artificial evolution experiments, artificial selection in agriculture, and introducing the concept of MLS. By applying these evidence‐based pedagogical practices, instructors will have the tools to prepare the next generation to use the fundamental evolutionary theory of MLS and apply its logic across diverse contexts.

### 4.7 Future Directions

By analyzing commonly used textbooks, this study represents an initial description of how multilevel selection is taught in undergraduate biology courses and finds that MLS is dismissed as unimportant. While textbooks can form the outline of course syllabi and influence the topics instructors cover, material covered in a course extends beyond assigned textbook readings. To more fully understand how multilevel selection is taught at the undergraduate level, future work should include observations of courses, analysis of assignments and examinations, and surveying instructors' and students' understanding and perceptions of MLS (see Yaworsky et al. 2015 for a survey of anthropologists). Additional work is also needed to understand effective strategies for presenting the contentious debate surrounding MLS to students in ways that promote student development of critical thinking skills. To supplement the missing MLS content and examples in textbooks, the creation of free MLS lecture slides and active learning modules would help instructors offer a more modern understanding of this fundamental concept in Evolutionary Biology.

## Author Contributions

**Charlotte A. Greene:** conceptualization (equal), data curation (lead), formal analysis (lead), visualization (equal), writing – original draft (lead), writing – review and editing (equal). **Sarah J. McPeek:** conceptualization (equal), data curation (lead), formal analysis (equal), methodology (equal), visualization (equal), writing – original draft (equal), writing – review and editing (equal). **Lisa Mitchem:** conceptualization (equal), data curation (equal), formal analysis (equal), writing – original draft (supporting), writing – review and editing (equal). **Anne B. Clark:** conceptualization (equal), data curation (equal), formal analysis (supporting), methodology (supporting). **Vincent A. Formica:** conceptualization (equal), data curation (supporting), formal analysis (supporting), writing – original draft (supporting), writing – review and editing (equal). **Conner S. Philson:** conceptualization (equal), data curation (equal), writing – original draft (equal), writing – review and editing (equal). **Robin A. Costello:** conceptualization (lead), data curation (equal), formal analysis (equal), investigation (lead), project administration (lead), writing – original draft (equal), writing – review and editing (equal).

## Conflicts of Interest

The authors declare no conflicts of interest.

## Supporting information


**Appendix S1:** ece372493‐sup‐0001‐AppendixS1.docx.

## Data Availability

All data are published in the University of Virginia data repository (DOI: https://doi.org/10.18130/V3/GFZMXN).

## References

[ece372493-bib-0001] Allen, D. , and K. Tanner . 2002. “Approaches to Cell Biology Teaching: Questions About Questions.” Cell Biology Education 1: 63–67. 12459794 10.1187/cbe.02-07-0021 PMC128545

[ece372493-bib-0003] Ballen, C. J. , and H. W. Greene . 2017. “Walking and Talking the Tree of Life: Why and How to Teach About Biodiversity.” PLoS Biology 15: e2001630. 28319149 10.1371/journal.pbio.2001630 PMC5358732

[ece372493-bib-0004] Bellamy, C. , K. Boughey , C. Hawkins , et al. 2020. “A Sequential Multi‐Level Framework to Improve Habitat Suitability Modelling.” Landscape Ecology 35: 1001–1020.

[ece372493-bib-0005] Beniermann, A. , L. Mecklenburg , and A. Upmeier zu Belzen . 2021. “Reasoning on Controversial Science Issues in Science Education and Science Communication.” Education in Science 11: 522.

[ece372493-bib-0006] Bentley, M. , C. Yates , J. Hein , G. Preston , and K. Foster . 2022. “Pleiotropic Constraints Promote the Evolution of Cooperation in Cellular Groups.” PLoS Biology 20: e3001626. 35658016 10.1371/journal.pbio.3001626 PMC9166655

[ece372493-bib-0007] Bergstrom, C. T. , and L. A. Dugatkin . 2023. Evolution. 3rd ed. WW Norton and Company.

[ece372493-bib-0008] Bijma, P. , and D. K. Aanen . 2010. “Assortment, Hamilton's Rule and Multilevel Selection.” Proceedings of the Royal Society B: Biological Sciences 277: 673–675. 10.1098/rspb.2009.1093 PMC2842734 19906665

[ece372493-bib-0009] Bijma, P. , and M. J. Wade . 2008. “The Joint Effects of Kin, Multilevel Selection and Indirect Genetic Effects on Response to Genetic Selection.” Journal of Evolutionary Biology 21: 1175–1188. 18547354 10.1111/j.1420-9101.2008.01550.x

[ece372493-bib-0011] Blackstone, N. W. , S. R. Blackstone , and A. T. Berg . 2020. “Variation and Multilevel Selection of SARS‐CoV‐2.” Evolution 74: 2429–2434. 32880957 10.1111/evo.14080 PMC7461403

[ece372493-bib-0013] Capp, J.‐P. , J. DeGregori , A. M. Nedelcu , et al. 2021. “Group Phenotypic Composition in Cancer.” Elife 10: e63518. 33784238 10.7554/eLife.63518 PMC8009660

[ece372493-bib-0014] Chitty, D. 1996. Do Lemmings Commit Suicide?: Beautiful Hypotheses and Ugly Facts. Oxford University Press.

[ece372493-bib-0015] Chuang, J. S. , O. Rivoire , and S. Leibler . 2009. “Simpson's Paradox in a Synthetic Microbial System.” Science 323: 272–275. 19131632 10.1126/science.1166739

[ece372493-bib-0016] Costello, R. A. , P. A. Cook , E. D. Brodie III , and V. A. Formica . 2023. “Multilevel Selection on Social Network Traits Differs Between Sexes in Experimental Populations of Forked Fungus Beetles.” Evolution 77: 289–303. 36622695 10.1093/evolut/qpac012

[ece372493-bib-0017] Craig, J. V. , and W. M. Muir . 1996. “Group Selection for Adaptation to Multiple‐Hen Cages: Beak‐Related Mortality, Feathering, and Body Weight Responses.” Poultry Science 75: 294–302. 10.3382/ps.0750294 8778719

[ece372493-bib-0018] Crowe, A. , C. Dirks , and M. P. Wenderoth . 2008. “Biology in Bloom: Implementing Bloom's Taxonomy to Enhance Student Learning in Biology.” CBE—Life Sciences Education 7: 368–381. 19047424 10.1187/cbe.08-05-0024 PMC2592046

[ece372493-bib-0019] Darwin, C. 1871. The Descent of Man, and Selection in Relation to Sex. D. Appleton.

[ece372493-bib-0020] Davies, N. B. , J. R. Krebs , and S. A. West . 2012. An Introduction to Behavioral Ecology, 4th Edition. Wiley‐Blackwell.

[ece372493-bib-0021] Diez Roux, A. V. 2004. “The Study of Group‐Level Factors in Epidemiology: Rethinking Variables, Study Designs, and Analytical Approaches.” Epidemiologic Reviews 26: 104–111. 15234951 10.1093/epirev/mxh006

[ece372493-bib-0022] Donohue, K. 2004. “Density‐Dependent Multilevel Selection in the Great Lakes Sea Rocket.” Ecology 85: 180–191.

[ece372493-bib-0023] Dugatkin, L. 2020. Principles of Animal Behavior. 4th ed. University of Chicago Press.

[ece372493-bib-0024] Eldakar, O. T. , and D. S. Wilson . 2011. “Eight Criticisms Not to Make About Group Selection.” Evolution 65: 1523–1526. 21644945 10.1111/j.1558-5646.2011.01290.x PMC3110649

[ece372493-bib-0025] Eldakar, O. T. , D. S. Wilson , M. J. Dlugos , and J. W. Pepper . 2010. “The Role of Multilevel Selection in the Evolution of Sexual Conflict in the Water Strider *aquarius remigis*: Role of Multilevel Selection in Sexual Conflict.” Evolution 64: 3183–3189. 20636357 10.1111/j.1558-5646.2010.01087.x PMC2962763

[ece372493-bib-0026] Ellen, E. D. , J. Visscher , J. A. M. van Arendonk , and P. Bijma . 2008. “Survival of Laying Hens: Genetic Parameters for Direct and Associative Effects in Three Purebred Layer Lines.” Poultry Science 87: 233–239. 10.3382/ps.2007-00374 18212365

[ece372493-bib-0027] Emerson, A. E. 1939. “Populations of Social Insects.” Ecological Monographs 9: 287–300.

[ece372493-bib-0028] Erdos, Z. , D. J. Studholme , M. D. Sharma , D. Chandler , C. Bass , and B. Raymond . 2024. “Manipulating Multi‐Level Selection in a Fungal Entomopathogen Reveals Social Conflicts and a Method for Improving Biocontrol Traits.” PLoS Pathogens 20: e1011775. 38527086 10.1371/journal.ppat.1011775 PMC10994555

[ece372493-bib-0100] Formica, V. A. , J. W. McGlothlin , C. W. Wood , et al. 2011. “Phenotypic Assortment Mediates the Effect of Social Selection in a Wild Beetle Population.” Evolution 65, no. 10: 2771–2781. 10.1111/j.1558-5646.2011.01340.x. 21967420

[ece372493-bib-0030] Freeman, S. , S. L. Eddy , M. McDonough , et al. 2014. “Active Learning Increases Student Performance in Science, Engineering, and Mathematics.” Proceedings of the National Academy of Sciences 111: 8410–8415. 10.1073/pnas.1319030111 PMC4060654 24821756

[ece372493-bib-0031] Freeman, S. , and J. C. Herron . 2013. Evolutionary Analysis. 5th Edition. Pearson.

[ece372493-bib-0032] Futuyma, D. , and M. Kirkpatrick . 2023. Evolution. 5th ed. Oxford University Press.

[ece372493-bib-0033] Gardner, A. 2015. “The Genetical Theory of Multilevel Selection.” Journal of Evolutionary Biology 28: 305–319. 25475922 10.1111/jeb.12566 PMC4415573

[ece372493-bib-0034] Goodnight, C. J. 2015. “Multilevel Selection Theory and Evidence: A Critique of Gardner, 2015.” Journal of Evolutionary Biology 28: 1734–1746. 26265012 10.1111/jeb.12685

[ece372493-bib-0035] Goodnight, C. J. , J. M. Schwartz , and L. Stevens . 1992. “Contextual Analysis of Models of Group Selection, Soft Selection, Hard Selection, and the Evolution of Altruism.” American Naturalist 140: 743–761.

[ece372493-bib-0036] Griesemer, J. R. , and M. J. Wade . 1988. “Laboratory Models, Causal Explanation and Group Selection.” Biology and Philosophy 3: 67–96.

[ece372493-bib-0037] Hanisch, S. , and D. Eirdosh . 2020. “Educational Potential of Teaching Evolution as an Interdisciplinary Science.” Evolution: Education and Outreach 13: 25.

[ece372493-bib-0038] Heisler, I. L. , and J. Damuth . 1987. “A Method for Analyzing Selection in Hierarchically Structured Populations.” American Naturalist 130: 582–602.

[ece372493-bib-0039] Hertler, S. C. , A. J. Figueredo , and M. Peñaherrera‐Aguirre . 2020. Multilevel Selection: Theoretical Foundations, Historical Examples, and Empirical Evidence. Springer International Publishing.

[ece372493-bib-0040] Kerr, B. , C. Neuhauser , B. J. M. Bohannan , and A. M. Dean . 2006. “Local Migration Promotes Competitive Restraint in a Host–Pathogen ‘Tragedy of the Commons’.” Nature 442: 75–78. 16823452 10.1038/nature04864

[ece372493-bib-0041] Kjelvik, M. K. , and E. H. Schultheis . 2019. “Getting Messy With Authentic Data: Exploring the Potential of Using Data From Scientific Research to Support Student Data Literacy.” CBE Life Sciences Education 18: es2. 31074698 10.1187/cbe.18-02-0023 PMC6755219

[ece372493-bib-0042] Laiolo, P. , and J. R. Obeso . 2012. “Multilevel Selection and Neighbourhood Effects From Individual to Metapopulation in a Wild Passerine.” PLoS One 7: e38526. 22745665 10.1371/journal.pone.0038526 PMC3380010

[ece372493-bib-0043] Lean, C. , and A. Plutynski . 2016. “The Evolution of Failure: Explaining Cancer as an Evolutionary Process.” Biology and Philosophy 31: 39–57.

[ece372493-bib-0044] Lion, S. , V. A. A. Jansen , and T. Day . 2011. “Evolution in Structured Populations: Beyond the Kin Versus Group Debate.” Trends in Ecology & Evolution 26: 193–201. 21353325 10.1016/j.tree.2011.01.006

[ece372493-bib-0045] Lobato, E. J. , and C. Zimmerman . 2019. “Examining How People Reason About Controversial Scientific Topics.” Thinking & Reasoning 25: 231–255.

[ece372493-bib-0046] Maher, B. 2009. The Nail in the Coffin for Group Selection? Nature, Advance Online Publication. 10.1038/nature08071.

[ece372493-bib-0047] Marín, C. , C. S. Philson , O. T. Eldakar , A. B. Clark , and M. J. Wade . 2025. “Abundant Empirical Evidence of Multilevel Selection Revealed by a Bibliometric Review.” Evolution in Prep.

[ece372493-bib-0048] Marshall, J. A. R. 2011. “Group Selection and Kin Selection: Formally Equivalent Approaches.” Trends in Ecology & Evolution 26: 325–332. 21620513 10.1016/j.tree.2011.04.008

[ece372493-bib-0049] McCartney, M. , R. Gonzalez , J. Colon , et al. 2025. “What Is a Biology Degree Without a Career Goal or a Strategy to Reach That Goal? An Analysis of Career Goals of Graduating Biology Majors.” Journal of College Science Teaching 54: 29–37.

[ece372493-bib-0050] McCauley, D. E. , and M. J. Wade . 1980. “Group Selection: The Genetic and Demographic Basis for the Phenotypic Differentiation of Small Populations of *Tribolium castaneum* .” Evolution 34: 813–821. 28563985 10.1111/j.1558-5646.1980.tb04020.x

[ece372493-bib-0051] McGlothlin, J. W. , A. J. Moore , J. B. Wolf , and E. D. Brodie III . 2010. “Interacting Phenotypes and the Evolutionary Process III. Social Evolution.” Evolution 64: 2558–2574. 20394666 10.1111/j.1558-5646.2010.01012.x

[ece372493-bib-0053] Muir, M. M. 2005. “Incorporation of Competitive Effects in Forest Tree or Animal Breeding Programs.” Genetics 170: 1247–1259. 15911590 10.1534/genetics.104.035956 PMC1451176

[ece372493-bib-0054] Muir, W. M. 1996. “Group Selection for Adaptation to Multiple‐Hen Cages: Selection Program and Direct Responses.” Poultry Science 75: 447–458. 10.3382/ps.0750447 8786932

[ece372493-bib-0055] Nonacs, P. , and K. M. Kapheim . 2012. “Modeling Disease Evolution With Multilevel Selection: HIV as a Quasispecies Social Genome.” Journal of Evolutionary Medicine 1: 235553.

[ece372493-bib-0056] Nordell, S. E. , and T. Valone . 2020. Animal Behavior: Concepts, Methods, and Applications. 3rd ed. Oxford University Press.

[ece372493-bib-0057] Okasha, S. 2006. Evolution and the Levels of Selection. Clarendon Press.

[ece372493-bib-0058] Okasha, S. 2010a. “Altruism Researchers Must Cooperate.” Nature 467: 653–655. 20930821 10.1038/467653a

[ece372493-bib-0059] Okasha, S. 2010b. “Levels of Selection.” Current Biology 20: R306–R307. 20392416 10.1016/j.cub.2010.01.025

[ece372493-bib-0060] Oulton, C. , J. Dillon , and M. M. Grace . 2004. “Reconceptualizing the Teaching of Controversial Issues.” International Journal of Science Education 26: 411–423.

[ece372493-bib-0061] Pepper, J. W. 2008. “Defeating Pathogen Drug Resistance: Guidance From Evolutionary Theory.” Evolution 62: 3185–3191. 18803686 10.1111/j.1558-5646.2008.00525.x

[ece372493-bib-0062] Philson, C. S. , J. G. A. Martin , and D. T. Blumstein . 2025. “Multilevel Selection on Individual and Group Social Behaviour in the Wild.” Proceedings of the Royal Society B 292: 20243061. 40101765 10.1098/rspb.2024.3061 PMC11919500

[ece372493-bib-0063] ProSocial World . n.d. “The Multilevel Selection Initiative.” https://www.prosocial.world/prosocial‐initiatives/the‐multilevel‐selection‐initiative.

[ece372493-bib-0064] Queller, D. C. 1992. “Quantitative Genetics, Inclusive Fitness, and Group Selection.” American Naturalist 139: 540–558.

[ece372493-bib-0065] Royle, N. J. , T. W. Pike , P. Heeb , H. Richner , and M. Kölliker . 2012. “Offspring Social Network Structure Predicts Fitness in Families.” Proceedings of the Biological Sciences 279: 4914–4922. 23097505 10.1098/rspb.2012.1701 PMC3497231

[ece372493-bib-0066] Rubenstein, D. R. 2022. Animal Behavior. 12th ed. Oxford University Press.

[ece372493-bib-0067] Saldaña, J. 2013. The Coding Manual for Qualitative Researchers. Sage.

[ece372493-bib-0068] Schroeder, C. M. , T. P. Scott , H. Tolson , T.‐Y. Huang , and Y.‐H. Lee . 2007. “A Meta‐Analysis of National Research: Effects of Teaching Strategies on Student Achievement in Science in the United States.” Journal of Research in Science Teaching 44: 1436–1460.

[ece372493-bib-0069] Schultheis, E. H. , M. K. Kjelvik , J. Snowden , L. Mead , and M. A. M. Stuhlsatz . 2023. “Effects of Data Nuggets on Student Interest in STEM Careers, Self‐Efficacy in Data Tasks, and Ability to Construct Scientific Explanations.” International Journal of Science and Mathematics Education 21: 1339–1362.

[ece372493-bib-0070] Searcy, C. A. , L. N. Gray , P. C. Trenham , and H. B. Shaffer . 2014. “Delayed Life History Effects, Multilevel Selection, and Evolutionary Trade‐Offs in the California Tiger Salamander.” Ecology 95: 68–77. 24649647 10.1890/13-0120.1

[ece372493-bib-0071] Secules, S. , C. McCall , J. A. Mejia , et al. 2021. “Positionality Practices and Dimensions of Impact on Equity Research: A Collaborative Inquiry and Call to the Community.” Journal of Engineering Education 110: 19–43.

[ece372493-bib-0072] Skinner, D. , and B. Howes . 2013. “The Required Textbook Friend or Foe? Dealing With the Dilemma.” Journal of College Teaching & Learning (TLC) 10: 133–142.

[ece372493-bib-0073] Smith, J. M. 1964. “Group Selection and Kin Selection.” Nature 201: 1145–1147.

[ece372493-bib-0074] Sober, E. , and D. S. Wilson . 1998. Unto Others: The Evolution and Psychology of Unselfish Behavior. Harvard University Press.

[ece372493-bib-0075] Stevens, L. , C. J. Goodnight , and S. Kalisz . 1995. “Multilevel Selection in Natural Populations of *Impatiens capensis* .” American Naturalist 145: 513–526.

[ece372493-bib-0076] Tanner, K. D. 2013. “Structure Matters: Twenty‐One Teaching Strategies to Promote Student Engagement and Cultivate Classroom Equity.” CBE Life Sciences Education 12: 322–331. 24006379 10.1187/cbe.13-06-0115 PMC3762997

[ece372493-bib-0077] Theobald, E. J. , M. J. Hill , E. Tran , et al. 2020. “Active Learning Narrows Achievement Gaps for Underrepresented Students in Undergraduate Science, Technology, Engineering, and Math.” Proceedings of the National Academy of Sciences of the United States of America 117: 6476–6483. 32152114 10.1073/pnas.1916903117 PMC7104254

[ece372493-bib-0078] Timmerman, B. E. , D. C. Strickland , and S. M. Carstensen . 2008. “Curricular Reform and Inquiry Teaching in Biology: Where Are Our Efforts Most Fruitfully Invested?” Integrative and Comparative Biology 48: 226–240. 21669786 10.1093/icb/icn064

[ece372493-bib-0080] Wade, M. J. 1980. “Group Selection, Population Growth Rate, and Competitive Ability in the Flour Beetles, *Tribolium* spp.” Ecology 61: 1056–1064.

[ece372493-bib-0081] Wade, M. J. , P. Bijma , E. D. Ellen , and W. Muir . 2010. “Group Selection and Social Evolution in Domesticated Animals.” Evolutionary Applications 3: 453–465. 25567938 10.1111/j.1752-4571.2010.00147.x PMC3352501

[ece372493-bib-0082] Wade, M. J. , and D. E. McCauley . 1980. “Group Selection: The Phenotypic and Genotypic Differentiation of Small Populations.” Evolution 34: 799–812. 28563979 10.1111/j.1558-5646.1980.tb04019.x

[ece372493-bib-0083] Wade, M. J. , D. S. Wilson , C. Goodnight , et al. 2010. “Multilevel and Kin Selection in a Connected World.” Nature 463: E8–E9. 20164866 10.1038/nature08809 PMC3151728

[ece372493-bib-0084] Warfa, A.‐R. M. 2016. “Mixed‐Methods Design in Biology Education Research: Approach and Uses.” CBE Life Sciences Education 15: rm5. 27856556 10.1187/cbe.16-01-0022 PMC5132391

[ece372493-bib-0085] Weinig, C. , J. A. Johnston , C. G. Willis , and J. N. Maloof . 2007. “Antagonistic Multilevel Selection on Size and Architecture in Variable Density Settings.” Evolution 61: 58–67. 17300427 10.1111/j.1558-5646.2007.00005.x

[ece372493-bib-0086] West, S. A. , A. S. Griffin , and A. Gardner . 2007. “Social Semantics: Altruism, Cooperation, Mutualism, Strong Reciprocity and Group Selection.” Journal of Evolutionary Biology 20: 415–432. 17305808 10.1111/j.1420-9101.2006.01258.x

[ece372493-bib-0087] Wild, G. , A. Gardner , and S. A. West . 2009. “Adaptation and the Evolution of Parasite Virulence in a Connected World.” Nature 459: 983–986. 19474791 10.1038/nature08071

[ece372493-bib-0088] Wild, G. , A. Gardner , and S. A. West . 2010. “Wild, Gardner & West Reply.” Nature 463: E9–E10.

[ece372493-bib-0089] Williams, G. C. 1966. Adaptation and Natural Selection: A Critique of Some Current Evolutionary Thought. Princeton University Press.

[ece372493-bib-0090] Wilson, D. S. 1975. “A Theory of Group Selection.” Proceedings of the National Academy of Sciences of the United States of America 72: 143–146. 1054490 10.1073/pnas.72.1.143 PMC432258

[ece372493-bib-0091] Wilson, D. S. 1979. “Structured Demes and Trait‐Group Variation.” American Naturalist 113: 606–610.

[ece372493-bib-0092] Wilson, D. S. 2008. “Truth and Reconciliation for Group Selection I: Why It Is Needed.” Huffington Post.

[ece372493-bib-0093] Wolf, J. B. , E. D. Brodie III , and A. J. Moore . 1999. “Interacting Phenotypes and the Evolutionary Process. II. Selection Resulting From Social Interactions.” American Naturalist 153: 254–266. 10.1086/303168 29585974

[ece372493-bib-0094] Wynne‐Edwards, V. C. 1962. Animal Dispersion in Relation to Social Behavior. Hafner.

[ece372493-bib-0095] Yaworsky, W. , M. Horowitz , and K. Kickham . 2015. “Gender and Politics Among Anthropologists in the Units of Selection Debate.” Biological Theory 10: 145–155.

[ece372493-bib-0096] Zeller, K. A. , T. W. Vickers , H. B. Ernest , and W. M. Boyce . 2017. “Multi‐Level, Multi‐Scale Resource Selection Functions and Resistance Surfaces for Conservation Planning: Pumas as a Case Study.” PLoS One 12: e0179570. 28609466 10.1371/journal.pone.0179570 PMC5469479

[ece372493-bib-0097] Zimmer, C. 2013. The Tangled Bank: An Introduction to Evolution. 2nd ed. Macmillan.

[ece372493-bib-0098] Zimmer, C. , and D. J. Emlen . 2019. Evolution: Making Sense of Life. 3rd ed. Roberts.

