# Supplementary material for: Multilevel Selection in the Margins: A Review of Its Representation in Undergraduate Biology Textbooks
Source: Ecol Evol. 2025 Nov 16;15(11):e72493. doi: 10.1002/ece3.72493 (PMC12620055; doi:10.1002/ece3.72493)
Supplement: Supplementary file 1 — Appendix S1: ece372493‐sup‐0001‐AppendixS1.docx. [file ECE3-15-e72493-s001.docx]

### **Appendix for Multilevel selection in the margins: A review of its representation in undergraduate biology textbooks**

| **Authors** | **Title and edition** | **Provided definition of natural selection** | **Allows for multiple levels of selection (Y/N)** |
| --- | --- | --- | --- |
| C. Bergstrom & L. Dugatkin | Evolution 3rd ed. | “Evolution by natural selection is the inevitable consequence if three conditions are met: variation, differential reproductive success, and inheritance.” | N |
| D. Futuyma & M. Kirkpatrick | Evolution 5th ed. | “Natural selection is any consistent difference in fitness among different classes of biological entities.” | Y |
| D. Emlen & C. Zimmer | Evolution: Making Sense of life 3rd ed. | “Natural selection is a mechanism that can lead to evolution, whereby differential survival and reproduction causes some genetic types to replace/outcompete others.” | Y |
| S. Freeman & J. Herron | Evolutionary Analysis 5th ed. | “[Darwin's] theory of evolution by natural selection, which he laid out in his introduction to On the Origin of Species, can be stated as a short set of postulates and a consequence that follows if the postulates are true… 1) The individuals in a population differ from one another. 2) the differences are, at least in part, passed from parents to offspring. 3) Some individuals are more successful at surviving and reproducing than others. 4) The successful individuals are not merely lucky; instead, they succeed because of the variant traits they have inherited and will pass to their offspring.” | N |
| C. Zimmer | The Tangled Bank: An Introduction to Evolution 2nd ed. | “The process by which individuals better adapted for their way of life in their environment preferentially survive to leave more offspring with their traits to future generations.” | N |
| S. E. Nordell & T. Valone | Animal Behavior: concepts, methods, and applications 3rd ed. | “Natural selection is a process in which some individuals in a population survive and reproduce, while others do not.” | N |
| L. Dugatkin | Principles of Animal Behavior 4th ed. | “The process of natural selection requires three prerequisites to be met: 1) variation in the trait - different varieties of the trait. 2) fitness consequences of the trait - different varieties of the trait must affect reproductive success and/or longevity differently. 3) A mode of inheritance - a means by which the trait is passed down to the next generation.” | Y |
| D. Rubenstein | Animal Behavior 12th ed. | “The logic of natural selection is such that evolutionary change is inevitable if just three conditions are met:1. Variation, with members of a population differing in a particular characteristic.2. Differential reproductive success, with some individuals with particular characteristics having more offspring than others.3. Heredity, with parents able to pass on those characteristics to their offspring.” | N |
| N. Davies, J. Krebs, & S. West | An Introduction to Behavioral Ecology 4th ed. | "(Darwin's) Theory of natural selection can be summarized as follows: 1) individuals within a species differ in their morphology, physiology, and behavior. 2) some of this variation is heritable. 3) Organisms have a huge capacity for increase in numbers. 4) As a result of this competition, some variants will leave for more offspring than others.” | N |

**Appendix Table 1.** Definitions of natural selection across textbooks.


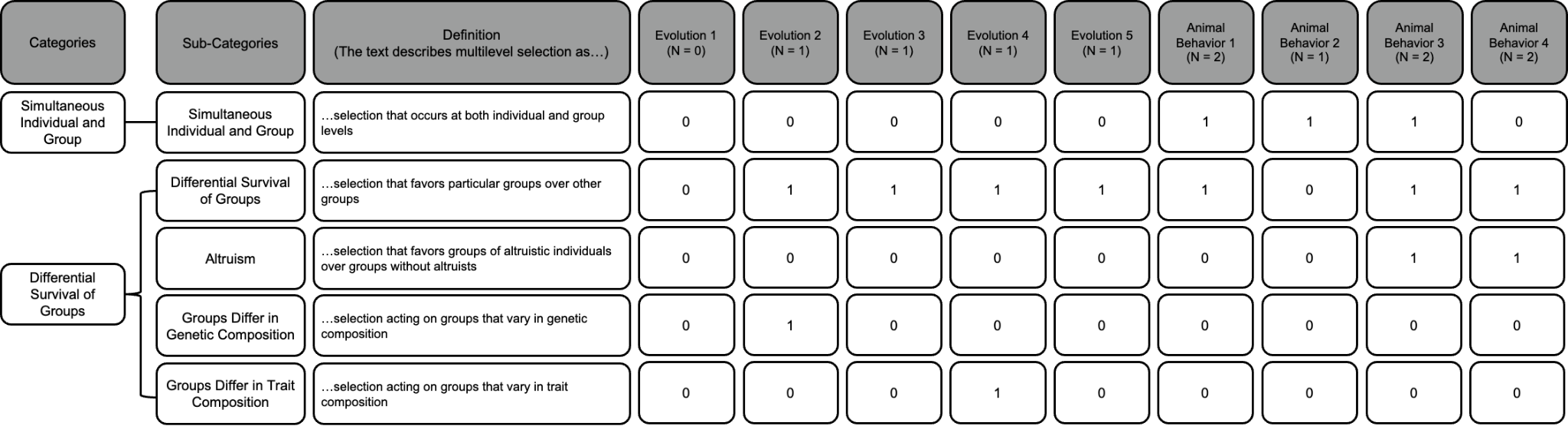


**Appendix Figure 1.** Codebook of how each textbook defines multilevel selection.


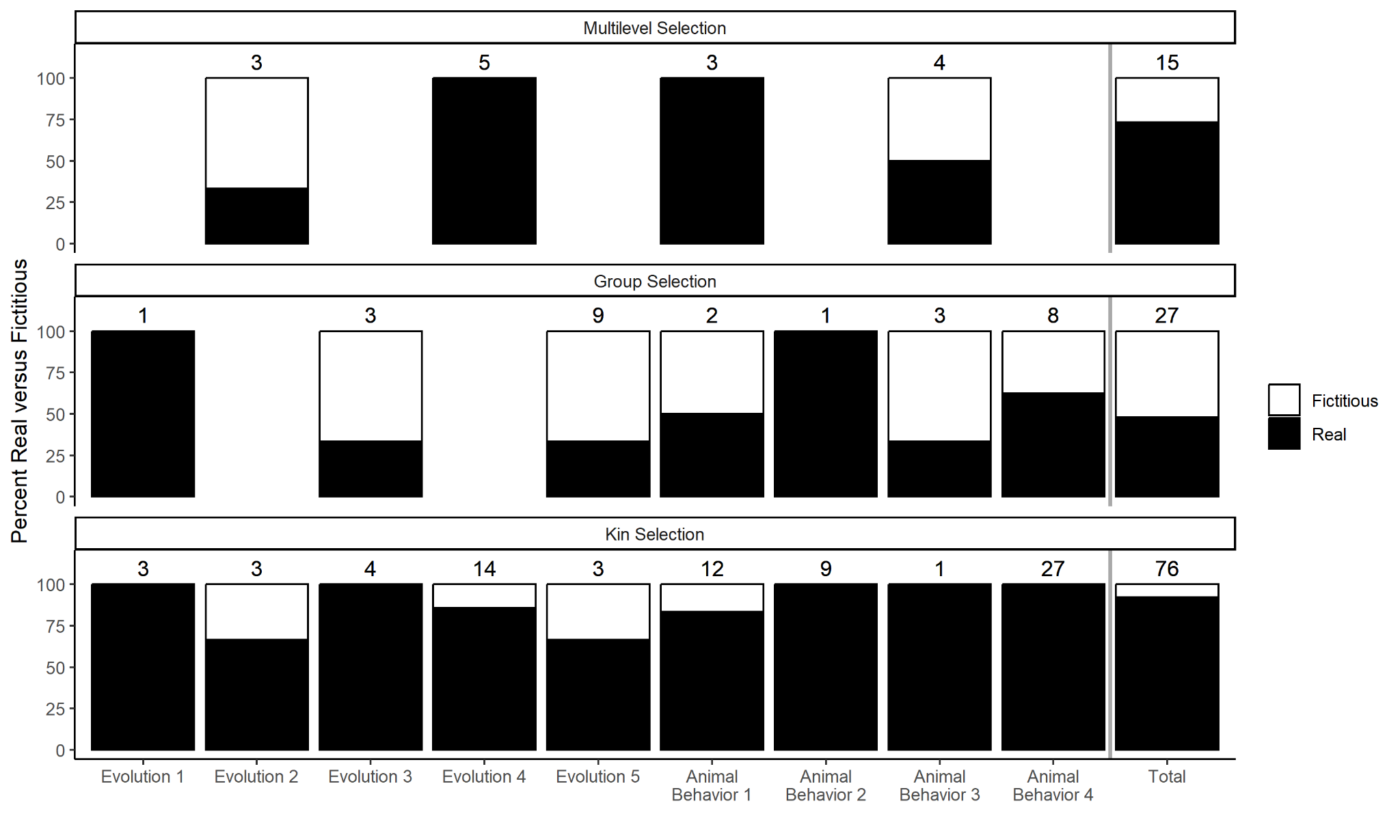


**Appendix Figure 2.** Proportion of fictitious versus real-world examples provided when discussing three different keywords (“multilevel selection”, “group selection”, and “kin selection”) across all textbooks included in our sample.


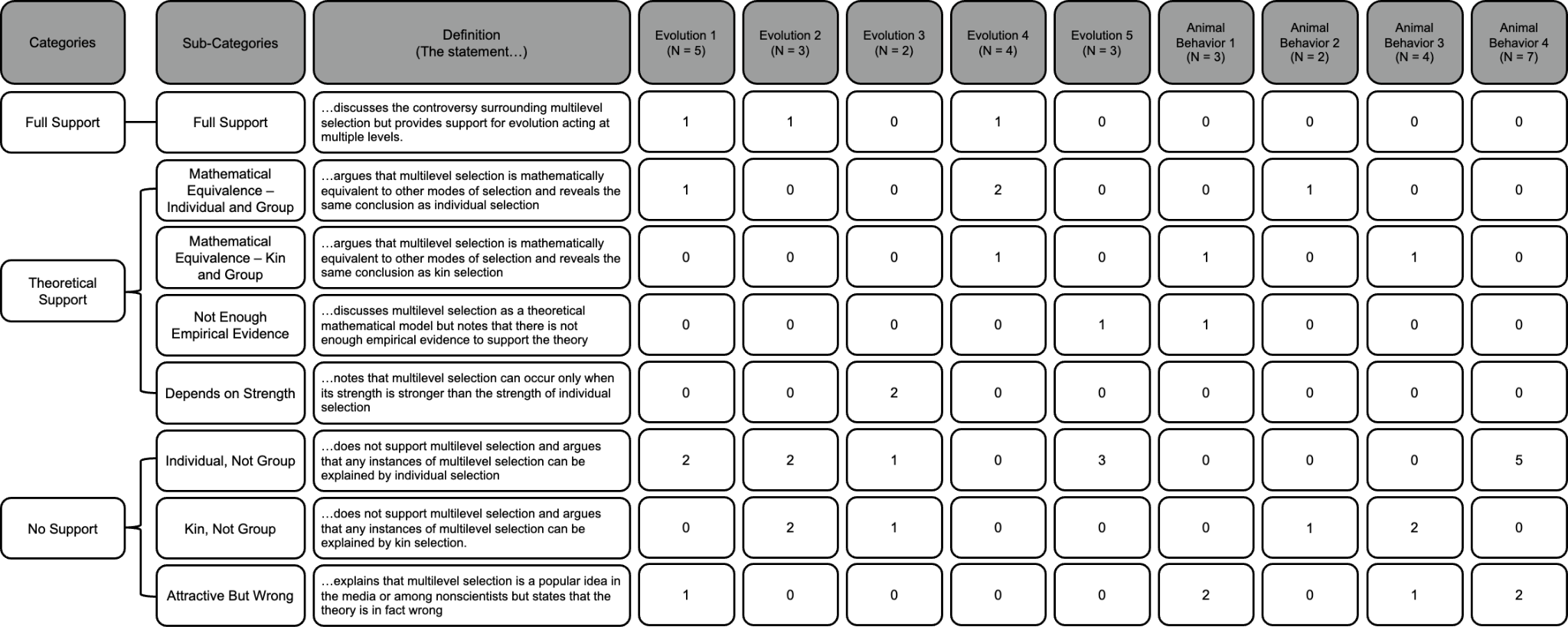


**Appendix Figure 3.** Codebook of how passages in each textbook discuss the controversy surrounding multilevel selection. Note that some textbooks simultaneously contain passages that fully support multilevel selection and passages that dismiss multilevel selection.


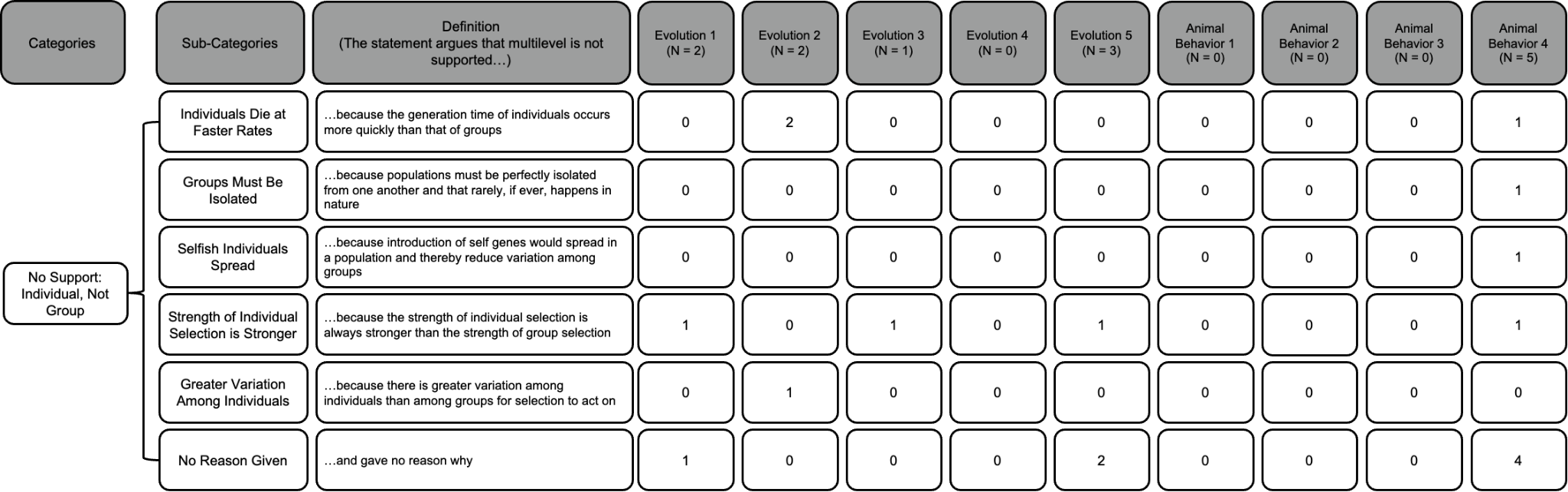


**Appendix Figure 4.** Codebook of the different reasons provided by each textbook for why selection occurs at the level of the individual, not the level of the group. Note that many textbooks provide multiple reasons for why selection occurs at the individual level but not at the group level.
